# TCR hypervariable regions expressed by T cells that respond to effective tumor vaccines

**DOI:** 10.1007/s00262-012-1217-5

**Published:** 2012-02-21

**Authors:** Kimberly R. Jordan, Jonathan D. Buhrman, Jonathan Sprague, Brandon L. Moore, Dexiang Gao, John W. Kappler, Jill E. Slansky

**Affiliations:** 1 Integrated Department of Immunology, School of Medicine, University of Colorado Denver, 1400 Jackson Street, Room K511, Denver, CO 80206 USA; 2 School of Public Health, University of Colorado Denver, Denver, CO 80045 USA; 3 Howard Hughes Medical Institute, National Jewish Health, Denver, CO 80206 USA

**Keywords:** Peptide vaccines, T cell repertoire, Tumor antigens, Peptide variants

## Abstract

A major goal of immunotherapy for cancer is the activation of T cell responses against tumor-associated antigens (TAAs). One important strategy for improving antitumor immunity is vaccination with peptide variants of TAAs. Understanding the mechanisms underlying the expansion of T cells that respond to the native tumor antigen is an important step in developing effective peptide-variant vaccines. Using an immunogenic mouse colon cancer model, we compare the binding properties and the TCR genes expressed by T cells elicited by peptide variants that elicit variable antitumor immunity directly ex vivo. The steady-state affinity of the natural tumor antigen for the T cells responding to effective peptide vaccines was higher relative to ineffective peptides, consistent with their improved function. Ex vivo analysis showed that T cells responding to the effective peptides expressed a CDR3β motif, which was also shared by T cells responding to the natural antigen and not those responding to the less effective peptide vaccines. Importantly, these data demonstrate that peptide vaccines can expand T cells that naturally respond to tumor antigens, resulting in more effective antitumor immunity. Future immunotherapies may require similar stringent analysis of the responding T cells to select optimal peptides as vaccine candidates.

**Electronic supplementary material:**

The online version of this article (doi:10.1007/s00262-012-1217-5) contains supplementary material, which is available to authorized users.

## Introduction

Increased frequencies of cytotoxic T lymphocytes (CTLs), which recognize antigenic peptides from tumors presented by major histocompatibility (MHC) class I molecules, correlate with improved cancer patient survival [1]. CTLs and the effector molecules they produce are critical in the elimination of cancer cells [2, 3]; however, they are also subject to regulatory mechanisms. Tumor-specific CTLs often recognize peptides derived from tumor-associated antigens (TAAs). One important strategy for improving antitumor immunity and overcoming these self-tolerance mechanisms is vaccination with peptide variants of TAAs (also known as mimotopes, heteroclitic peptides, peptide analogues, or altered peptide ligands) that improve binding to either MHC molecules [4] or tumor-specific T cell receptor (TCR) molecules [5, 6]. These vaccines stimulate the expansion of the often low-affinity TAA-specific T cells that escape negative selection during development [7, 8] and are suboptimally activated by native tumor antigens [9, 10]. Clinical trials have shown increased frequencies of TAA-specific T cells following vaccination with peptide variants, demonstrating the potential of antigen-specific immunotherapy [11].

Peptide variants are most often selected as vaccine candidates because they bind with stronger affinity or stimulate TAA-specific T cells more effectively than the native tumor antigen in vitro. Paradoxically, vaccination with these variants often elicits T cells with diminished antitumor activity relative to T cells that naturally respond to native tumor antigens [12–15]. Using the CT26 transplantable tumor model [7, 16], we previously showed that vaccination with variants of the immunodominant peptide, gp70_423–431_ (AH1), elicited variable antitumor immunity [17, 18]. These peptides robustly stimulated the tumor-specific T cell clone used to identify the variants and elicited more tumor-specific T cells from the endogenous repertoire of BALB/c mice than the native AH1 peptide [17, 18]. However, vaccination with these peptides elicited a range of antitumor immunity. The ineffective peptides elicited T cells that failed to kill AH1-loaded target cells or produce IFNγ after stimulation with the native tumor antigen [17]. Surprisingly, these functionally deficient T cells produced IFNγ after stimulation with the variant peptide used in the vaccine, suggesting that the ineffective peptide variants were not antagonists or partial agonists, as in other systems [19].

Understanding the mechanisms underlying the expansion of T cells that respond poorly to native tumor antigens is an important step in developing effective peptide-variant vaccines. The repertoire of T cells responding to peptide variants is often different than the repertoire of T cells responding to native tumor antigens [10, 12, 20, 21]. As addressed in this study, we hypothesized that the effective peptide variants likely elicited a different repertoire of tumor-specific T cells that expressed TCRs with higher affinity for the native tumor antigen. Since in vitro expansion of tumor antigen-specific T cells can dramatically skew T cell cultures in favor of higher-affinity T cell clones [22], we compared the binding properties and the TCR genes expressed by T cells elicited by peptide variants directly ex vivo. The T cells elicited by an effective peptide-variant bound fluorescent MHC-tetramers containing the native tumor antigen with a higher staining intensity. These T cells did not cross-react with an ineffective peptide variant, suggesting that the repertoires of T cells responding to these variant vaccines were different and did not overlap. In addition, the effective peptide variants elicited a repertoire of T cells that was closely related in sequence to the T cells elicited by the native TAA, expressing a common Jβ sequence and a common CDR3β motif, and the frequency of sequences containing this CDR3β motif correlated with tumor protection. Thus, the effective peptide-variant vaccines enhanced the expansion of T cells that respond to the native TAA, rather than activating a new subset of T cells. Importantly, these results demonstrate that T cells responding to native tumor antigens can be effectively stimulated to prevent tumor growth, provided they are activated through the TCR by the appropriate signal. The implication of these results is that the most effective peptide vaccines may be identified by measuring the responses of tumor antigen-specific T cells found in patients, rather than isolated T cell clones.

## Materials and methods

### Mice

Six- to eight-week-old female BALB/cAnNCr mice were purchased from the National Cancer Institute/Charles River Laboratories. All animal protocols were reviewed and approved by the Institutional Animal Care and Use Committee of National Jewish Health.

### Cells

Sf9 and High Five insect cells ([23], Invitrogen) were cultured and infected as described [2, 17]. T cell clones were cultured as described [9]. The T cell lines were cloned by limiting dilution and expanded with 5 × 10^5^/ml irradiated CT26 tumor cells expressing the costimulatory molecule B7 for 2 weeks prior to flow cytometry and TCR sequence analysis.

### Peptides

βgal (TPHGAGRIL [24]), AH1 (SPSYVYHQF [16]), A5 (SPSYAYHQF [9]), 39 (MNKYAYHML [18]), 15 (MPKYAYHML [18]), WMF (SPTYAYWMF [2]), and F1A5 (FPSYAYHQF [17]) peptides were identified as described. Soluble synthetic peptides were ≥95% pure (Chi Scientific).

### Vaccination

Sf9 insect cells were infected with recombinant baculovirus (BV) encoding L^d^ and beta-2-microglobulin molecules covalently linked to peptides via a glycine-rich linker as described [2, 25].

### Antibodies and staining reagents

Preparation of the fluorescent L^d^ tetramer linked to the AH1 peptide using a disulfide trap or loaded with exogenous AH1 peptide has been described [17, 18]. Soluble TCRs containing CDR3β motifs were prepared by inserting Vβ8.3 and Vα6 gene segments expressed by the A5-4E11 or 39-1D4 T cell clones into a BV expression vector [2]. Purified TCR molecules were conjugated to fluorescent streptavidin molecules as described and used to stain insect cells expressing membrane-bound peptide-MHC molecules [2]. The median fluorescent intensity (MFI) of L^d^ high cells was determined. Antibodies specific for the L^d^ molecule (28.14.8S), CD8 (53–6.7, Southern Biotech), IFNγ (XMG1.2, eBioscience), B220 (RA3-6B2, BD Pharmigen), CD4 (RM4-5, BD Pharmigen), and MHC-II (M5/114.15.2, BD Pharmigen) were used for flow cytometric analyses. The B220, CD4, and MHC-II antibodies were analyzed in the “dump gate” for the tetramer, IFNγ, and Vβ staining experiments. For costaining experiments, splenocytes from vaccinated mice were stained with L^d^-tet linked to the AH1 peptide with a disulfide trap and conjugated to steptavidin-PE for 2 h at 37°C to facilitate internalization and limit competition between tetramers and L^d^-tet linked to the F1A5 or WMF peptides conjugated to streptavidin-AF647 were added and stained for an additional 2 h at 4°C. All samples were run on a BD FACSCaliber flow cytometer.

### Intracellular cytokine staining

Splenocytes were stimulated with the indicated peptide for 5 h in the presence of monensin, a protein transport inhibitor (GolgiStop, BD Cytofix/Cytoperm Plus Fixation/Permeabilization Kit, BD Pharmingen), fixed, permeabilized, and stained as described [17].

### Tetramer decay and titration

For the decay assay, 2 × 10^6^ splenocytes were stained in 96-well plates with L^d^-tet loaded with the AH1 peptide for 1 h at RT. The AH1 tetramer with the disulfide trap was not used in these experiments because its fluorescence intensity is not reduced during the timeframe of this experiment. The cells were washed extensively and resuspended in FACS buffer (1x phosphate-buffered saline, 1× HEPES buffer, 0.1% sodium azide, 2% fetal bovine serum) and 100 μg/ml F(ab)’ fragments of the L^d^-specific antibody (28.14.8S). Approximately 2 × 10^5^ cells were removed at each time point and fixed in 1% paraformaldehyde. Total fluorescence (*T*_f_) was determined using the following formula [26]:

$$
\begin{aligned}
T_{\text{f}} =& \left[ {\left( { {\text{\% of AH1-tet}}^{+}
{\text{of CD8}}^{+} {\text{ cells}}} \right)\left( {{\text{MFI of AH1-tet}}^{+} {\text{ population}}} \right)}
\right]_{\text{peptide\,vaccine}}\\& -\left[ {\left( { {\text{\% of
 AH1-tet}}^{+} {\text{ of CD}}8^{+} {\text{ cells}}} \right)\left(
{{\text{MFI of AH1-tet}}^{+} {\text{ population}}} \right)}
\right]_{{\beta {\text{gal vaccine}}}}
\end{aligned}
$$

Normalized fluorescence (*N*_f_) was calculated by dividing the *T*_f_ at each time point by the *T*_f_ at time 0. The natural log (ln) of the *N*_f_ was plotted versus time and the data for each mouse were fit to a one-phase exponential decay curve [(*y* = *y*0*e(−*kt*) where *y* = ln *N*_f_, *k* = decay constant, and *t* = time)] using Prism version 4.0, Graphpad software. The half-lives were calculated for each mouse [(half-life = −ln(2)/*k*)] and compared using an unpaired two-tailed *t* test.

For the tetramer titration assay, splenocytes were stained as above for 2 h at 4°C. The MFI was calculated using the following formula [26]: MFI = (*T*_f_)/(% of AH1-tet^+^ of CD8^+^ cells). The MFI was plotted versus the concentration of tetramer and fit to a one-site binding curve [*Y* = (Bmax*X)/(*K*_D_ + *X*)] using Prism version 4.0, Graphpad software. The *K*_D_ values for each mouse were compared using an unpaired two-tailed *t* test.

### TCR sequencing

Approximately 1 × 10^5^ CD8^+^ AH1-tet^+^ splenocytes were separated with a MoFlo^®^ High-Performance Cell Sorter. RNA was extracted using the RNeasy Mini Kit (Qiagen), and cDNA was synthesized using the QuantiTect Reverse Transcription Kit (Qiagen) according to the manufacturer’s instructions. For the plasmid sequencing method, the cDNA was PCR-amplified using the primer sets described in Supplemental Table 1 [27–29]. Amplified DNA was cloned into the pCR2.1 vector using the TOPO TA Cloning Kit (Invitrogen), and sequences were determined using an internal Cβ primer (Supplemental Table 1) and capillary DNA sequencing instruments (ABI 3730s). For the high-throughput method, the cDNA was PCR-amplified for 15 cycles using a forward primer specific for all Vβ8 family members containing an adaptor sequence (in bold) and an internal multiplex identifier (MID) sequence (in italics, Supplemental Table 1) and a reverse primer specific for Cβ containing a different adaptor sequence (in bold, Supplemental Table 1). PCR fragments were separated by gel electrophoresis, purified using the Gel Extraction Kit (Qiagen), and further amplified 30 cycles using primers specific for the adaptor sequences. After gel extraction and quantification, the PCR products were combined and subjected to high-throughput sequencing as previously described [30]. Sequences from both methods were analyzed using a computer software program developed by our laboratory that uses a BLAST-type algorithm to identify Vβ and Jβ gene sequence information [28, 31]. This program translated and aligned the sequences, identified the MID sequences and sorted the results according to each vaccine, distinguished the germline-encoded and randomized CDR3β region of each sequence, calculated the length of each CDR3β region, and determined the number of sequences containing the shared CDR3β motif. The sequences of the TCRs expressed by the T cell clones (Fig. 6a) were determined by directly sequencing PCR products amplified from their cDNA using the Vβ8.3 and Cβ primers or Vα6 primers.

## Results

### Vaccination with the F1A5 peptide elicits T cells with higher affinity for the AH1 peptide

Previously identified peptide variants effectively stimulated a tumor antigen-specific T cell clone both in vitro and in vivo, but elicited variable antitumor responses from the endogenous T cell repertoire [17]. Vaccination with the F1A5 peptide variant protected 90% of mice from tumor growth, while tumors grew in all of the mice vaccinated with the WMF peptide [2, 17]. The increased tumor protection afforded by the F1A5 peptide was attributed to the expansion of more tumor-specific T cells that exhibited effector function after stimulation with the AH1 peptide, although the mechanism for this differential expansion of functional T cells was not determined [17].

**Fig. 1:**
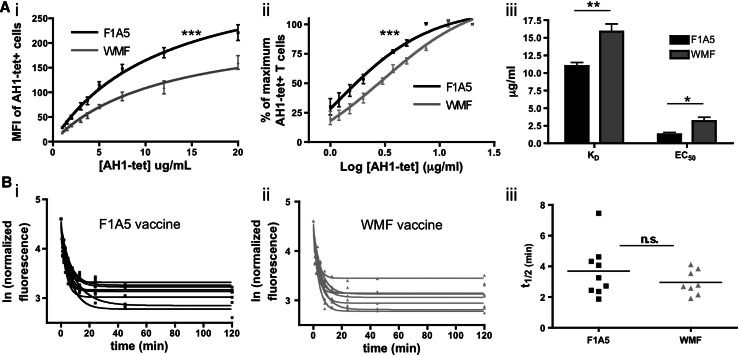
T cells elicited by peptide-variant vaccines have different affinity for the tumor antigen. **a** (*i*) Splenocytes from mice vaccinated with the indicated peptides were stained with antibodies specific for dump, CD8, and LFA-1 molecules and a titration of L^d^-tet loaded with the AH1 peptide. The MFI of AH1-tet^+^ CD8^+^ LFA-1^+^ cells was graphed after subtracting the fluorescence of tetramer-binding cells in mice vaccinated with the irrelevant βgal peptide and fit to a one-site binding curve. *Error bars* represent the SEM (*n* = 6 mice). Curves were compared using an *F* test (***p < 0.0001). (*ii*) Cells from (**a**i) were graphed as a percentage of the maximum number of AH1-tet-binding cells at each concentration of tetramer and fit to a sigmoidal dose–response curve. Curves were compared using an *F* test (***p < 0.0001). (*iii*) K_D_ and EC_50_ values were determined for individual mice using one-site binding curves or sigmoidal dose–response curves as in (**a**i) and (**a**ii) and compared using a Student’s *t* test (**p = 0.002, *p = 0.0097, *n* = 6 mice). **b** Splenocytes from (*i*) F1A5- or (*ii*) WMF-vaccinated mice were stained as in (**a**). The total fluorescence of the dump^−^ CD8^+^ LFA-1^+^ AH1-tet^+^ cells was determined at the indicated time. The natural log (ln) of the normalized fluorescence was graphed and fit to an exponential decay curve. (*iii*) The half-life of AH1-tet staining (*t*_1/2_) was determined for each mouse and compared using a Student’s *t* test (n.s. = not significant)

When factors such as CD8 and TCR expression levels are considered, the relative median fluorescence intensity (MFI) of multimerized peptide-MHC (tetramer) staining reflects the binding affinity of T cells [26, 32–35]. Although direct affinity measurements would be ideal, this cannot be performed with current technology on multiclonal T cells ex vivo. We hypothesized that the WMF peptide was less effective because the T cells responding to this vaccine bound to the AH1–L^d^ complex with lower affinity. Thus, we measured the MFI of tetramer staining of splenocytes from mice vaccinated with a previously described insect cell vaccine expressing either an effective (F1A5) or ineffective (WMF) variant peptide [2, 17]. We detected differences in the relative MFI of the AH1-tetramer^+^ T cells (Fig. 1ai). After normalizing for the differences in the number of AH1-specific T cells (Supplemental Fig. 1a), the F1A5-elicited T cells required a lower concentration of tetramer for half of the maximum number of tetramer^+^ cells to bind the AH1-tetramer (Fig. 1aii). Using both the binding affinities and the EC_50_ values from these data curves, we determined that the F1A5-elicited T cells have a higher affinity for the AH1 tumor antigen than the WMF-elicited T cells, consistent with our hypothesis (Fig. 1aiii). These differences may be underestimated due to the limitations of analog instruments and the loss of linearity at higher fluorescence intensities. Due to the limited number of AH1-specific cells in AH1-vaccinated mice relative to background tetramer staining in βgal-vaccinated mice, we were not able to reliably determine the KD or EC_50_ of tetramer binding for these cells (Supplemental Fig 1b).

Since tetramer-dissociation rates correlate with peptide-MHC/TCR affinities in some systems [26], we measured the half-life (*t*_1/2_) of tetramer binding to the responding T cell repertoire (Fig. 1b). The average *t*_1/2_ of T cells from F1A5- and WMF-vaccinated mice was not statistically different (Fig. 1biii). Although many factors contribute to *K*_d_ values calculated using multivalent interactions [35], these observed avidity differences may be due to faster on-rates rather than longer half-lives [36]. In summary, the F1A5-elicited T cells bound to the AH1 antigen with increased affinity, which may explain the previously described improved effector function exhibited by T cells responding to this peptide vaccine [17].

### Few tumor-specific T cells cross-react with both the F1A5 and WMF peptides

**Fig. 2:**
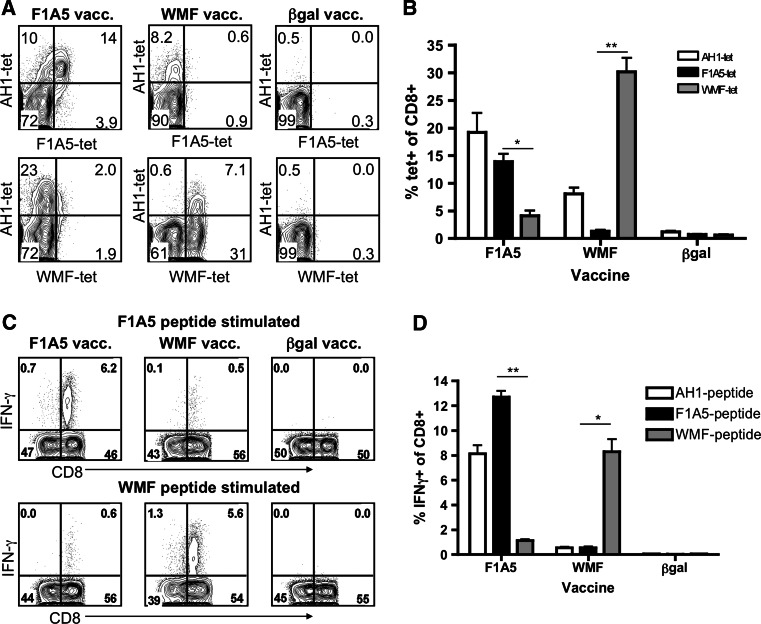
The repertoire of T cells elicited by the variant peptides F1A5 and WMF is distinct and few cells overlap. **a** Splenocytes from mice vaccinated with the indicated peptide were stained and gated on CD8^+^ dump^−^ cells. Staining with the L^d^-tet linked to the AH1 peptide (*y*-axis) and L^d^-tet linked to the F1A5 or WMF peptides (*x*-axis) is shown. The *dot plots* are representative of 3 independent experiments. **b** Splenocytes from multiple mice were stained as in (**a**), and the percentage of tet^+^ cells was determined by histogram analysis (*n* = 6 mice). *Error bars* represent the SEM, and groups were compared by an unpaired two-tailed *t* test (*p = 0.0002, **p < 0.0001). **c** Splenocytes from (**a**) were assessed for intracellular IFNγ production after stimulation with the indicated peptides by staining with antibodies specific for dump, CD8, and IFNγ molecules. The events shown are representative dot plots of 2 independent experiments. **d** T cells from multiple mice were assessed for intracellular cytokine production as in (**c**) (*n* = 3 mice). Although not shown in (**c**), IFNγ production by splenocytes stimulated with the AH1 peptide was assessed using the same gating. *Error bars* represent the SEM, and groups were compared by an unpaired two-tailed *t* test (*p = 0.0017, **p < 0.0001)

Next, we determined the mechanism involved in the decreased affinity of the WMF-elicited T cells (Fig. 1) and their decreased effector responses to the AH1 peptide [17]. Diminished T cell responses may have been caused by incomplete activation of these cells or by the expansion of a different repertoire of T cells with lower affinity. To distinguish these possibilities, we determined whether the effective and ineffective peptide variants elicit overlapping repertoires of T cells. Splenocytes from vaccinated mice were co-stained with the AH1-tetramer and variant tetramers (Fig. 2a). It is intriguing that so few of these T cells cross-reacted with both peptide variants, since they were designed to stimulate the same AH1-specific T cell clone. However, even among the AH1-specific T cells expanded by the vaccines, few cells bound to both variant tetramers (Fig. 2a, b). Furthermore, the differences in the MFI of AH1-tetramer staining (described in Fig. 1) were also detected in this experiment and are shown in the dot plots (Fig. 2a).

In some systems, functional measurements more accurately assess the number of antigen-specific T cells [37]. To ensure the tetramer-binding experiments did not underestimate the number of T cells that cross-react with both peptide variants, we analyzed intracellular IFNγ production by splenocytes from vaccinated mice stimulated with either the F1A5 or the WMF peptides (Fig. 2c, d). Similar to the tetramer staining results, few T cells cross-reacted with both variant peptides. In addition, as previously reported [17], few WMF-elicited T cells produced IFNγ after stimulation with the AH1 peptide (Fig. 2d). Together, these data suggest that vaccination with the F1A5 and WMF peptides elicited different portions of the endogenous AH1-specific T cell repertoire.

### F1A5 and WMF peptides elicit different repertoires of T cells

To directly characterize the TCR gene usage of the AH1-specific T cells elicited by the peptide variants, we co-stained splenocytes from vaccinated mice with the AH1-tetramer and a panel of antibodies to the mouse TCR Vβ genes (Supplementary Fig. 2a). In addition to the F1A5 and WMF peptides, we also analyzed the response to the native tumor antigen and to three other previously characterized peptide variants, 15 (ineffective), and A5 and 39 (effective) [9, 17, 18]. All of these peptide variants were also selected using the Vβ8.3^+^ CT T cell clone and, not surprisingly, they elicited AH1-specific T cells that predominantly expressed the Vβ8 family of TCRs (Supplementary Fig. 2b).

**Fig. 3:**
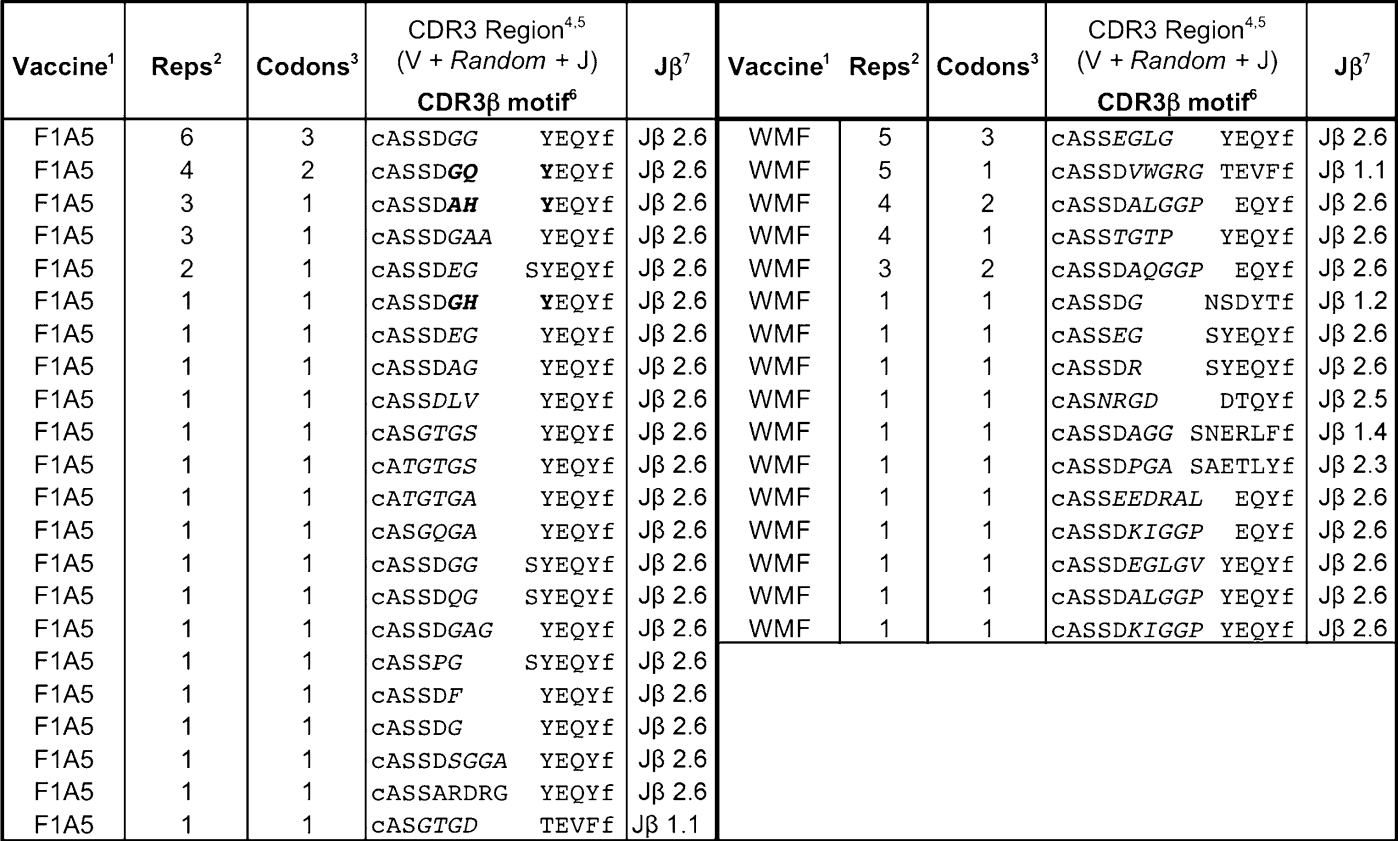
The AH1-specific T cells elicited by the F1A5 peptide express TCRs with the Vβ8.3 and Jβ2.6 gene segments and a common CDR3β motif, and are different from the T cells elicited by the WMF peptide. ^1^AH1-tet^+^ cells from the spleens of 5 mice vaccinated with the indicated peptide were pooled, separated by FACSorting, and the gene segments encoding Vβ8.3 TCRs were sequenced. ^2^Replicates: the number of analyzed sequences that encoded a particular amino acid sequence. ^3^Codons: the number of nucleotide sequences that encoded a particular amino acid sequence. ^4^The sequences were aligned using the shared cysteine residue in the Vβ sequence and the shared phenylalanine residue in the Jβ sequence. Germline-encoded sequences are shown as normal text, and sequence encoded by “n” nucleotide additions is shown in italics. *Capital letters* indicate the CDR3β loop. ^6^Sequences encoding the common CDR3β motif, consisting of a small hydrophobic residue, followed by a larger polar residue, followed by the tyrosine residue encoded by the Jβ2.6 gene segment, are shown in *bold*. ^7^The Jβ gene segments encoded by each sequence are listed

Since most of the T cells responding to these peptide vaccines expressed Vβ8 TCR genes, it was not clear whether the peptides elicited different repertoires of T cells. Thus, we analyzed the CDR3β and Jβ sequences expressed by AH1-specific T cells in mice vaccinated with the F1A5 and WMF peptides. The identified TCR sequences with in-frame gene rearrangements are listed (Fig. 3). Nearly all of the T cells elicited by the F1A5 peptide expressed TCRs with the Jβ2.6 gene segment (97%), in contrast to the WMF-elicited TCR sequences that encoded several different Jβ gene segments (72% Jβ2.6). While there was some junctional diversity in the F1A5-elicited TCR sequences, we identified a shared three amino acid motif in the predicted CDR3β loop that was absent from the WMF-elicited TCR sequences. This CDR3β motif consisted of a glycine or alanine residue, followed by a large polar residue and the tyrosine residue of Jβ2.6 (Fig. 3). The first two amino acids of the motif were not germline-encoded, but encoded by different frames of Dβ2 in combination with *N*-nucleotides. A second CDR3β pattern consisting of a glycine residue, followed by a glycine or alanine residue may also be unique to mice vaccinated with effective peptides. Strikingly, most of the TCRs used a Jβ2.6 segment that was truncated during rearrangement at its N-terminus precisely at the tyrosine residue. This tyrosine residue is unique to Jβ2.6 and is predicted to lie on the exposed portion of the CDR3β loop available for contact with peptide-MHC [38]. A second common feature of nearly all of the F1A5-elicited Vβ8.3^+^ T cells was a relatively short CDR3β length of 10 amino acids. Although the structural analysis of these interactions has yet to be performed, it is tempting to suggest that the short CDR3β loop and the tyrosine residue in the CDR3β motif are required for more stable interactions with the tyrosine residues at positions 4 or 6 of the AH1 peptide. These results are consistent with the affinity analyses and tetramer staining data and demonstrate that vaccination with the effective F1A5 and ineffective WMF peptides elicits different repertoires of AH1-specific T cells.

### Effective and ineffective peptides elicit different repertoires of AH1-specific T cells

**Fig. 4:**
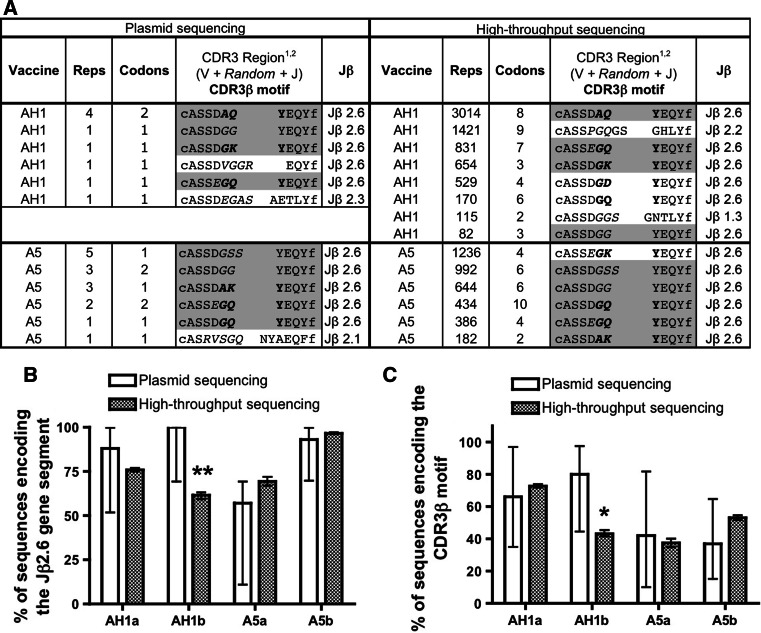
T cells elicited by the native tumor antigen are similar to those elicited by an effective peptide variant, and similar results are obtain with plasmid and high-throughput sequencing methods. **a** ^1^This figure is formatted as in Fig. 3. ^2^ *Gray* shaded regions indicate CDR3β sequences that were identified using both traditional sequencing methods of plasmids encoding the PCR-amplified region (*left*) or high-throughput sequencing of PCR products amplified from the same cDNA (*right*). **b** Vβ8.3 TCR sequences were amplified from the cDNA of AH1-specific cells isolated from vaccinated mice and sequenced using traditional sequencing methods (*white bars*) or high-throughput sequencing (*patterned bars*). The percentage of Vβ8.3 TCR sequences encoding the Jβ2.6 gene segment was calculated for each sample. *Error bars* represent the 95% confidence intervals (**p = 0.008). **c** As in (**b**), the average percentages of sequences encoding the common CDR3β motif were calculated for each sample. *Error bars* represent the 95% confidence intervals (*p = 0.017)

To determine whether the frequency and number of AH1-specific T cells expressing the CDR3β motif correlates with tumor protection, we extended the repertoire analysis to the responses elicited by the native tumor antigen and other previously identified peptide variants using two different techniques. We sorted AH1-specific T cells from individual vaccinated mice, generated cDNA, and performed PCR using Vβ8 and Cβ primers. Between 1,000 and 35,000, sequences were obtained per mouse using the Roche 454 Sequencing System and around 20 sequences were obtained per mouse using standard sequencing methods (Supplemental Table 1). We first verified that both techniques produce similar results by comparing the sequences obtained from the same preparation of cDNA using either method (Fig. 4). Both sequencing methods revealed that Vβ8.3^+^ T cells responding to the native tumor antigen express the CDR3β motif, similar to the T cells responding to an effective peptide variant, the A5 peptide. Many identical sequences were identified from the same cDNA sample (Fig. 4a) and the frequencies of sequences expressing the Jβ2.6 gene segment and the CDR3β motif for most of the samples were similar (Fig. 4b, c), demonstrating that standard sequencing methods and high-throughput sequencing give similar results.

**Fig. 5:**
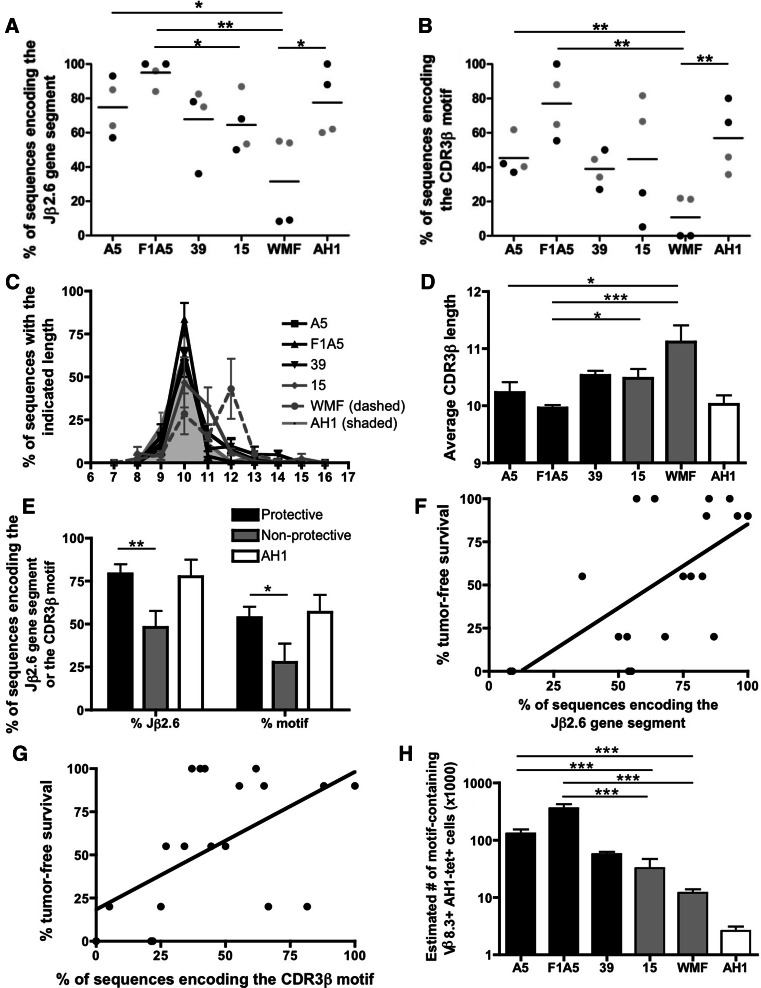
T cells elicited by effective peptide variants express Vβ8.3 TCRs with a CDR3β motif that correlates with tumor protection. **a** The average percentages of sequences encoding the Jβ2.6 gene segment were calculated for the sequences of the Vβ8.3-expressing TCRs from vaccinated mice (analyzed as in Fig. 4). *Symbols* represent individual mice analyzed by traditional sequencing methods (*black*) or high-throughput sequencing (*gray*). The* bar* indicates the mean, and groups were compared using a Student’s *t* test (*p < 0.05, **p < 0.009, *n* = 4 mice). **b** As in (**a**), the average percentages of sequences encoding the common CDR3β motif were calculated for each vaccine. **c** The number of amino acids encoded in the CDR3β loop of each Vβ8.3 sequence was determined. The percentage of sequences encoding the indicated CDR3β length was calculated for each mouse, as in traditional spectratyping analysis. The *errors bars* represent the SEM (*n* = 4 mice). **d** The average length of the CDR3β chains was determined as in (**c**) for the Vβ8.3 sequences and compared using a Student’s *t* test (*p < 0.05, ***p = 0.007, *n* = 4 mice). **e** The average percentage of sequences encoding the Jβ2.6 gene segment (*left*) or the CDR3β motif (*right*) was calculated for the effective (A5, F1A5, and 39 in *black*), ineffective (15 and WMF in *gray*), and native peptides (*white*). *Error bars* represent the SEM. Groups were compared using a Student’s *t* test (**p = 0.0073, *p = 0.039). **f** The frequency of sequences encoding the Jβ2.6 gene segment (*x*-axis, from a) was plotted versus the frequency of tumor-free survival observed for each vaccine (35), and a positive correlation was found using a Spearman’s nonparametric correlation test (*r* = 0.6256, *p* = 0.0032). **g** As in (**f**), the correlation of the frequency of sequences encoding the CDR3β motif (*x*-axis, from **b**) and tumor-free survival was analyzed (*r* = 0.5582, *p* = 0.0105). **h** The estimated number of AH1-tet^+^ T cells expressing Vβ8.3 TCRs with the CDR3β motif was determined by multiplying the average frequency of CDR3β motif-containing TCR sequences (from **b**) by the number of Vβ8.3^+^ AH1-tet^+^ cells in the spleens of a separate cohort of mice (frequencies from these mice are shown in Supplementary Fig. 1a) and compared using a Student’s *t* test (***p < 0.004)

Similar to the AH1-elicited TCR sequences, many of the Vβ8.3^+^ TCRs elicited by the effective A5, F1A5, and 39 peptides expressed the Jβ2.6 gene segment and the CDR3β motif (Fig. 5a, b). The distribution of CDR3β lengths expressed by T cells elicited by the effective peptides was also more similar to the AH1-elicited T cells than those elicited by the WMF peptide (Fig. 5c, d). Peptide 15 elicited a highly variable repertoire of T cells, consistent with its variable tumor protection and other previously measured T cell responses [17, 18]. These data suggest that the T cells elicited by the effective peptides and the native tumor antigen were related, expressing a heavily selected CDR3β motif, which may be involved in the specific recognition of the AH1–L^d^ complex.

**Fig. 6:**
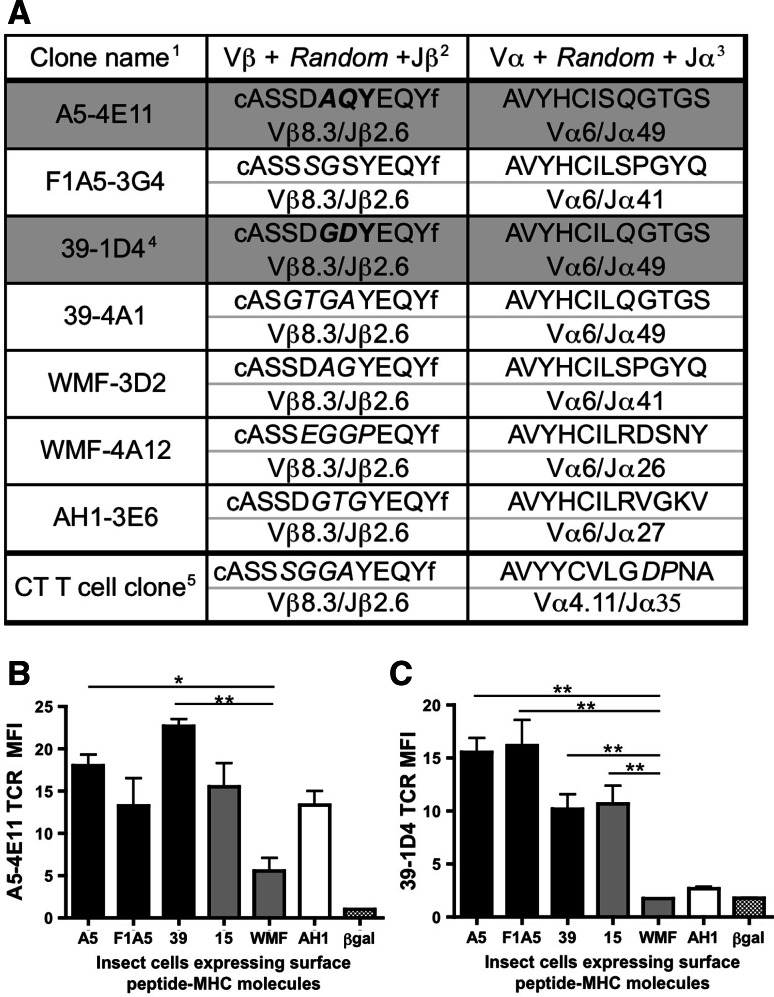
CDR3β motif-containing TCRs bind poorly to the WMF peptide. **a** ^1^T cell clones were screened for CD8^+^ AH1-tet^+^ Vβ8.3^+^ cells and expanded using irradiated CT26-B7 tumor cells. T cell clones were named after the vaccine used to generate the clone and the plate and well number they were located in. ^2^Sequences of the CDR3β region were determined after PCR amplification using Vβ- and Cβ-specific primers. ^3^Sequences of the CDR3a region were determined after PCR amplification using Vα- and Cα-specific primers. ^4^The cDNA sequence of 39-1D4 and A5-4E11 was subcloned into a protein expression vector for binding studies. ^5^The CT T cell clone was derived from a BALB/c mouse vaccinated with irradiated CT26 tumor cells expressing the cytokine GM-CSF (9). **b** Sequence encoding the TCRs derived from T cell clones expressing Vβ8.3 TCRs with the CDR3β motif (39-1D4 and A5-4E11) was inserted into a BV expression vector, and purified TCR protein was multimerized and conjugated to a fluorescent molecule. A5-4E11 (**b**) or 39-1D4 (**c**) TCR multimers and the L^d^ antibody 28.14.8 were used to stain insect cells infected with BV encoding the indicated peptide variant–L^d^ complex. The soluble TCR MFI was determined for insect cells expressing similar levels of L^d^ molecules (*n* = 3). Groups were compared using a Student’s *t* test (*p < 0.05, **p < 0.01)

Combining the data for the effective and ineffective peptides, we found a significant increase in the frequency of sequences encoding the Jβ2.6 gene segment and the CDR3β motif in TCRs elicited by the effective peptides (Fig. 5e). Furthermore, there was a statistically significant correlation between the frequency of both the Jβ2.6 gene segment and the CDR3β motif and tumor-free survival afforded by these peptide vaccines (Fig. 6f, g). Finally, we estimated the number of Vβ8.3^+^ T cells expressing the CDR3β motif in the spleens of vaccinated mice. Effective peptide vaccines have significantly more of these cells (Fig. 5h), suggesting that the increased frequency and number of Vβ8.3^+^ T cells expressing the CDR3β motif in mice vaccinated with effective peptide vaccines may contribute to effective antitumor immunity. We found similar trends among the Vβ8.1^+^ T cells (Supplemental Fig. 3), but not the Vβ8.2^+^ T cells (data not shown).

Surprisingly, although these junctional sequences were similar to that expressed by the CT T cell clone (Fig. 6a), we did not find the exact CT-TCR sequence in these mice or in the mice vaccinated with the peptide variants. The absence of the CT T cell clone in this study suggests that this clone is not representative of the endogenous repertoire of T cells responding to these vaccines and explains the lack of cross-reactivity between the F1A5- and WMF-elicited repertoires (Fig. 2). Perhaps the different mode of immunization (whole cell irradiated CT26-GM [9]) and the in vitro expansion used to generate the CT clone accounts for its dominance in those studies, and absence here.

### TCRs containing the CDR3β motif bind to the WMF peptide with lower affinity

To study the binding properties of the motif-containing T cells described above, we required both the β- and α-chain sequences of the TCRs identified in Figs. 3 and 4. We expanded and cloned AH1-specific T cells from vaccinated mice as described [9]. The TCR genes expressed by these T cell clones did not reflect the sequencing results we obtained from fresh uncultured splenocytes, particularly for the T cell clones from mice vaccinated with the ineffective peptide variants (Fig. 6a). However, we obtained several antigen-specific T cell clones that expressed TCRs containing the CDR3β motif and that had identical Vβ chains as some of those identified ex vivo. These T cell clones expressed a Vα6 TCR, distinct from the Vα4.11 TCR expressed by the CT T cell clone (Fig. 6a).

To determine the binding properties of TCRs containing the CDR3β motif, we subcloned the TCR molecules expressed by the A5-4E11 and the 39-1D4 T cell clones (Fig. 6a) into a BV expression vector. The sequences of these TCRs were highly selected in mice vaccinated with AH1, A5, and F1A5 peptides and not identified in mice vaccinated with the ineffective 15 and WMF peptides. We previously showed that the relative binding of multimeric-soluble TCRs to peptide–MHC complex expressed on BV-infected insect cells correlates with the affinity of monomeric TCR for the peptide–MHC complex determined by surface plasmon resonance [17, 18, 33, 39, 40]. Therefore, we stained insect cells expressing each of the peptide variant–L^d^ complexes with fluorescent multimeric TCRs derived from the A5-4E11 and 39-1D4 clones. The soluble TCRs bound to insect cells expressing the native tumor antigen, but not the irrelevant βgal peptide, demonstrating that these TCRs are specific for the AH1 peptide (Fig. 6b, c). The staining intensity of insect cells expressing the WMF peptide was significantly lower, indicating that this CDR3β motif-containing TCR does not bind efficiently to the WMF peptide (Fig. 6b). The binding of the 39-1D4 TCR more closely correlated with its representation in the ex vivo repertoire analysis, binding to the A5 and F1A5 peptides and not to the WMF peptide (Fig. 6c). The low-affinity interaction between the CDR3β motif-containing TCRs and the WMF peptide provides a simple mechanistic explanation for the absence of these cells following vaccination with the WMF peptide.

## Discussion

Although the peptide variants used in our study effectively stimulated a tumor-specific T cell clone in vitro, they elicited variable responses from the endogenous T cell population. In this study, we found that effective cancer peptide vaccines stimulate T cells with hypervariable regions similar to those that respond to the natural tumor antigen, not a de novo T cell repertoire. These results suggest that the heterogeneity in the repertoire of AH1-specific T cells contributes to the ineffectiveness of some of the peptide-variant vaccines.

Similar to our results, a study of the T cell response to the Melan-A/MART-1_26–35_ TAA showed that the TCRβ gene usage of the T cells responding to the native peptide was broader than that responding to the peptide variant [21]. They also identified a CDR3β motif that was shared among several patients, a so-called public TCR, although these motif-containing T cells were not among the dominant clones identified in each patient and did not have a higher functional avidity [21]. They concluded that the repertoires responding to the peptide variants and the native tumor antigen were subtly different but over-lapping, and that the observed functional differences were due to subtle structural changes in the TCR. In contrast, the T cells responding to the ineffective peptide variants in our study express a unique repertoire of TCRs that do not overlap with the repertoire responding to the native tumor antigen and that do not contain a significant fraction of the shared CDR3β motif. Therefore, in our study, only the effective peptide variants elicited a repertoire of T cells that over-lapped with those responding to the native tumor antigen.

The most effective peptide variants (A5 and F1A5) have minimal amino acid sequence changes relative to the AH1 peptide. It is, therefore, not surprising that these vaccines raise similar T cell repertoires. These data suggest that peptides with conservative amino acid changes may be more effective in stimulating antitumor immunity by eliciting a repertoire of T cells that mimics the response to the native tumor antigen. In agreement with this premise, another study concluded that substitutions at MHC-binding positions create immunogenic peptide variants with an overall similar structure to the native peptide [4]. However, the opposite has also been shown in these studies. Peptides specifically designed to encode conservative amino acid changes were ineffective vaccines and raised a repertoire of T cells different from that of the native tumor antigen [10, 20, 41]. These discrepancies suggest that even subtle amino acid substitutions may cause unpredictable changes in the repertoire and binding kinetics of the responding endogenous T cells.

The T cells responding to the F1A5 peptide express higher-affinity T cell receptors that functionally recognize the AH1 TAA better than the T cells responding to the ineffective peptide, WMF. Furthermore, soluble TCRs encoding the common CDR3β motif do not bind efficiently to the WMF peptide, providing a mechanistic explanation for the lack of these TCRs in the responding repertoire. We predict from these data that the structure of the WMF peptide prevents productive interactions with TCRs containing the common CDR3β motif. Specifically, the tryptophan residue at position 7 of the WMF peptide may affect the position of the adjacent tyrosine residue at position 6, precluding its predicted interaction with the tyrosine residue encoded by the Jβ2.6 gene segment of the motif-containing TCRs. Important for peptide vaccine development, these results imply that T cells responding to the ineffective variants (like WMF) cannot be “rescued” or forced to respond to the tumor antigen with better adjuvants; they are not anergic or improperly activated, they simply express T cell receptors that interact poorly with the native tumor antigen.

A clinically relevant strategy in the design of effective peptide vaccines is to optimize the T cells used in the identification of peptide variants. We selected our peptide variants based on improved binding to and activation of the CT clone, an AH1-specific T cell clone that was in vitro expanded and is not similar to those analyzed ex vivo (Fig. 6). The T cell clones that were found in the endogenous repertoire bound to the peptides that elicit better tumor-specific responses (Fig. 6). Therefore, we predict that peptide variants selected with an ex vivo tumor-specific repertoire, rather than an in vitro selected T cell clone, may be the most effective peptides for cancer vaccines***.***

Due to the unpredictable nature of the responding T cell repertoire, even if optimal T cells are used for peptide selection, ineffective peptides may be unavoidable in peptide-variant vaccine development. Therefore, a general strategy to improve the response to peptide variants such as the WMF peptide should be considered. In this particular case, rather than providing additional adjuvants or cytokines during T cell activation, the repertoire of the T cell response needs to be re-focused to those with high affinity for the native tumor antigen. Although strategies to engineer T cells with receptors that have high affinity for tumor antigens have been developed and employed in the clinical treatment for cancer [42, 43], effective antitumor immunity may be more easily achieved by “boosting” the peptide-variant responses with native tumor antigens [14, 44]. This strategy may increase the number of high-affinity T cells that cross-react with native tumor antigens and reduce competition with T cells that only bind to peptide variants.

In summary, peptide-variant vaccines improve the proliferation and activation of T cells that respond to native tumor antigens. Because the T cell response is unpredictable and different for every antigen, optimal peptide variants may be most successfully identified using a representative TAA-specific T cell population to select preferred residue changes from a large and random set of peptides.

### Electronic supplementary material

Below is the link to the electronic supplementary material.

Supplementary material 1 (DOCX 2155 kb)

## References

[1] Reynolds SR, Zeleniuch-Jacquotte A, Shapiro RL, Roses DF, Harris MN, Johnston D, Bystryn JC (2003). Vaccine-induced CD8^+^ T-cell responses to MAGE-3 correlate with clinical outcome in patients with melanoma. Clin Cancer Res.

[2] Jordan KR, McMahan RH, Oh JZ, Pipeling MR, Pardoll DM, Kedl RM, Kappler JW, Slansky JE (2008). Baculovirus-infected insect cells expressing peptide-MHC complexes elicit protective antitumor immunity. J Immunol.

[3] Dunn GP, Old LJ, Schreiber RD (2004). The three Es of cancer immunoediting. Annu Rev Immunol.

[4] van Stipdonk MJ, Badia-Martinez D, Sluijter M, Offringa R, van Hall T, Achour A (2009). Design of agonistic altered peptides for the robust induction of CTL directed towards H-2Db in complex with the melanoma-associated epitope gp100. Cancer Res.

[5] Kersh GJ, Miley MJ, Nelson CA, Grakoui A, Horvath S, Donermeyer DL, Kappler J, Allen PM, Fremont DH (2001). Structural and functional consequences of altering a peptide MHC anchor residue. J Immunol.

[6] Sharma AK, Kuhns JJ, Yan S, Friedline RH, Long B, Tisch R, Collins EJ (2001). Class I major histocompatibility complex anchor substitutions alter the conformation of T cell receptor contacts. J Biol Chem.

[7] McWilliams JA, Sullivan RT, Jordan KR, McMahan RH, Kemmler CB, McDuffie M, Slansky JE (2008). Age-dependent tolerance to an endogenous tumor-associated antigen. Vaccine.

[8] Colella TA, Bullock TN, Russell LB, Mullins DW, Overwijk WW, Luckey CJ, Pierce RA, Restifo NP, Engelhard VH (2000). Self-tolerance to the murine homologue of a tyrosinase-derived melanoma antigen: implications for tumor immunotherapy. J Exp Med.

[9] Slansky JE, Rattis FM, Boyd LF, Fahmy T, Jaffee EM, Schneck JP, Margulies DH, Pardoll DM (2000). Enhanced antigen-specific antitumor immunity with altered peptide ligands that stabilize the MHC-peptide-TCR complex. Immunity.

[10] Wang R, Wang-Zhu Y, Gabaglia CR, Kimachi K, Grey HM (1999). The stimulation of low-affinity, nontolerized clones by heteroclitic antigen analogues causes the breaking of tolerance established to an immunodominant T cell epitope. J Exp Med.

[11] Fourcade J, Kudela P, Andrade Filho PA, Janjic B, Land SR, Sander C, Krieg A, Donnenberg A, Shen H, Kirkwood JM, Zarour HM (2008). Immunization with analog peptide in combination with CpG and montanide expands tumor antigen-specific CD8^+^ T cells in melanoma patients. J Immunother.

[12] Hou Y, Kavanagh B, Fong L (2008). Distinct CD8^+^ T cell repertoires primed with agonist and native peptides derived from a tumor-associated antigen. J Immunol.

[13] Iero M, Squarcina P, Romero P, Guillaume P, Scarselli E, Cerino R, Carrabba M, Toutirais O, Parmiani G, Rivoltini L (2007). Low TCR avidity and lack of tumor cell recognition in CD8(+) T cells primed with the CEA-analogue CAP1-6D peptide. Cancer Immunol Immunother.

[14] Stuge TB, Holmes SP, Saharan S, Tuettenberg A, Roederer M, Weber JS, Lee PP (2004). Diversity and recognition efficiency of T cell responses to cancer. PLoS Med.

[15] Speiser DE, Baumgaertner P, Voelter V, Devevre E, Barbey C, Rufer N, Romero P (2008). Unmodified self antigen triggers human CD8 T cells with stronger tumor reactivity than altered antigen. Proc Natl Acad Sci USA.

[16] Huang AY, Gulden PH, Woods AS, Thomas MC, Tong CD, Wang W, Engelhard VH, Pasternack G, Cotter R, Hunt D, Pardoll DM, Jaffee EM (1996). The immunodominant major histocompatibility complex class I-restricted antigen of a murine colon tumor derives from an endogenous retroviral gene product. Proc Natl Acad Sci U S A.

[17] Jordan KR, McMahan RH, Kemmler CB, Kappler JW, Slansky JE (2010). Peptide vaccines prevent tumor growth by activating T cells that respond to native tumor antigens. Proc Natl Acad Sci U S A.

[18] McMahan RH, McWilliams JA, Jordan KR, Dow SW, Wilson DB, Slansky JE (2006). Relating TCR-peptide-MHC affinity to immunogenicity for the design of tumor vaccines. J Clin Invest.

[19] Sloan-Lancaster J, Allen PM (1996). Altered peptide ligand-induced partial T cell activation: molecular mechanisms and role in T cell biology. Annu Rev Immunol.

[20] Le Gal FA, Ayyoub M, Dutoit V, Widmer V, Jager E, Cerottini JC, Dietrich PY, Valmori D (2005). Distinct structural TCR repertoires in naturally occurring versus vaccine-induced CD8^+^ T-cell responses to the tumor-specific antigen NY-ESO-1. J Immunother.

[21] Wieckowski S, Baumgaertner P, Corthesy P, Voelter V, Romero P, Speiser DE, Rufer N (2009). Fine structural variations of alphabetaTCRs selected by vaccination with natural versus altered self-antigen in melanoma patients. J Immunol.

[22] Dietrich PY, Walker PR, Schnuriger V, Saas P, Perrin G, Guillard M, Gaudin C, Caignard A (1997). TCR analysis reveals significant repertoire selection during in vitro lymphocyte culture. Int Immunol.

[23] Crawford F, Huseby E, White J, Marrack P, Kappler JW (2004). Mimotopes for alloreactive and conventional T Cells in a peptide-MHC display library. PLoS Biol.

[24] Gavin MA, Gilbert MJ, Riddell SR, Greenberg PD, Bevan MJ (1993). Alkali hydrolysis of recombinant proteins allows for the rapid identification of class I MHC-restricted CTL epitopes. J Immunol.

[25] Jordan KR, Crawford F, Kappler JW, and Slansky JE (2009) Vaccination of mice with baculovirus-infected insect cells expressing antigenic proteins. Current protocols in immunology/edited by John E Coligan et al. [Chapter 2:Unit 2 15] 10.1002/0471142735.im0215s85 PMC3343717 19347845

[26] Savage PA, Boniface JJ, Davis MM (1999). A kinetic basis for T cell receptor repertoire selection during an immune response. Immunity.

[27] Pannetier C, Cochet M, Darche S, Casrouge A, Zoller M, Kourilsky P (1993). The sizes of the CDR3 hypervariable regions of the murine T-cell receptor beta chains vary as a function of the recombined germ-line segments. Proc Natl Acad Sci USA.

[28] Arden B, Clark SP, Kabelitz D, Mak TW (1995). Mouse T-cell receptor variable gene segment families. Immunogenetics.

[29] Lefranc MP, Giudicelli V, Ginestoux C, Bodmer J, Muller W, Bontrop R, Lemaitre M, Malik A, Barbie V, Chaume D (1999). IMGT, the international ImMunoGeneTics database. Nucl Acids Res.

[30] Wang C, Sanders CM, Yang Q, Schroeder HW, Wang E, Babrzadeh F, Gharizadeh B, Myers RM, Hudson JR, Davis RW, Han J (2010). High throughput sequencing reveals a complex pattern of dynamic interrelationships among human T cell subsets. Proc Natl Acad Sci U S A.

[31] Barth RK, Kim BS, Lan NC, Hunkapiller T, Sobieck N, Winoto A, Gershenfeld H, Okada C, Hansburg D, Weissman IL (1985). The murine T-cell receptor uses a limited repertoire of expressed V beta gene segments. Nature.

[32] Wooldridge L, Lissina A, Cole DK, van den Berg HA, Price DA, Sewell AK (2009). Tricks with tetramers: how to get the most from multimeric peptide-MHC. Immunology.

[33] Crawford F, Kozono H, White J, Marrack P, Kappler J (1998). Detection of antigen-specific T cells with multivalent soluble class II MHC covalent peptide complexes. Immunity.

[34] Yee C, Savage PA, Lee PP, Davis MM, Greenberg PD (1999). Isolation of high avidity melanoma-reactive CTL from heterogeneous populations using peptide-MHC tetramers. J Immunol.

[35] Fahmy TM, Bieler JG, Edidin M, Schneck JP (2001). Increased TCR avidity after T cell activation: a mechanism for sensing low-density antigen. Immunity.

[36] Govern CC, Paczosa MK, Chakraborty AK, Huseby ES (2010). Fast on-rates allow short dwell time ligands to activate T cells. Proc Natl Acad Sci U S A.

[37] McWilliams JA, McGurran SM, Dow SW, Slansky JE, Kedl RM (2006). A modified tyrosinase-related protein 2 epitope generates high-affinity tumor-specific T cells but does not mediate therapeutic efficacy in an intradermal tumor model. J Immunol.

[38] Gras S, Kjer-Nielsen L, Burrows SR, McCluskey J, Rossjohn J (2008). T-cell receptor bias and immunity. Curr Opin Immunol.

[39] Huseby ES, Crawford F, White J, Marrack P, Kappler JW (2006). Interface-disrupting amino acids establish specificity between T cell receptors and complexes of major histocompatibility complex and peptide. Nat Immunol.

[40] White J, Crawford F, Fremont D, Marrack P, Kappler J (1999). Soluble class I MHC with beta2-microglobulin covalently linked peptides: specific binding to a T cell hybridoma. J Immunol.

[41] Cole DK, Edwards ES, Wynn KK, Clement M, Miles JJ, Ladell K, Ekeruche J, Gostick E, Adams KJ, Skowera A, Peakman M, Wooldridge L, Price DA, Sewell AK (2010). Modification of MHC anchor residues generates heteroclitic peptides that alter TCR binding and T cell recognition. J Immunol.

[42] Morgan RA, Dudley ME, Wunderlich JR, Hughes MS, Yang JC, Sherry RM, Royal RE, Topalian SL, Kammula US, Restifo NP, Zheng Z, Nahvi A, de Vries CR, Rogers-Freezer LJ, Mavroukakis SA, Rosenberg SA (2006). Cancer regression in patients after transfer of genetically engineered lymphocytes. Science.

[43] Bobisse S, Rondina M, Merlo A, Tisato V, Mandruzzato S, Amendola M, Naldini L, Willemsen RA, Debets R, Zanovello P, Rosato A (2009). Reprogramming T lymphocytes for melanoma adoptive immunotherapy by T-cell receptor gene transfer with lentiviral vectors. Cancer Res.

[44] Bolonaki I, Kotsakis A, Papadimitraki E, Aggouraki D, Konsolakis G, Vagia A, Christophylakis C, Nikoloudi I, Magganas E, Galanis A, Cordopatis P, Kosmatopoulos K, Georgoulias V, Mavroudis D (2007). Vaccination of patients with advanced non-small-cell lung cancer with an optimized cryptic human telomerase reverse transcriptase peptide. J Clin Oncol.

