# Supplementary material for: TCR hypervariable regions expressed by T cells that respond to effective tumor vaccines
Source: Cancer Immunol Immunother. 2012 Feb 21;61(10):1627–38. doi: 10.1007/s00262-012-1217-5 (PMC3410973; doi:10.1007/s00262-012-1217-5)
Supplement: Supplementary file 1 — Supplementary material 1 (DOCX 2155 kb) [file 262_2012_1217_MOESM1_ESM.pdf]

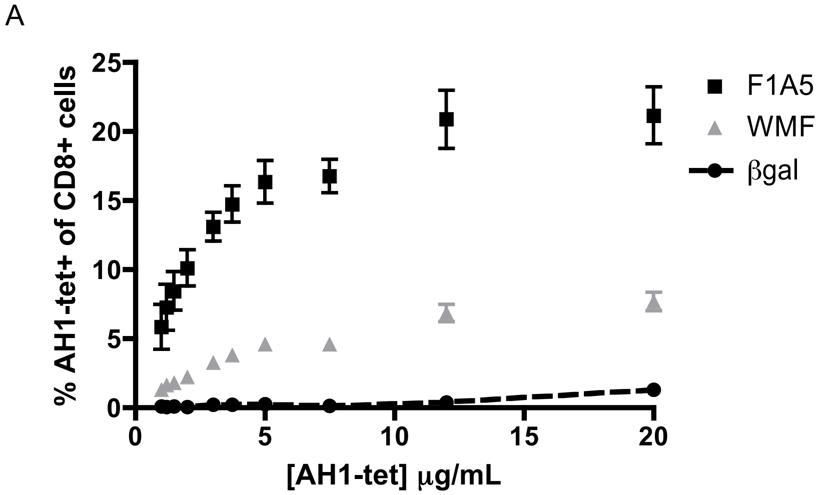

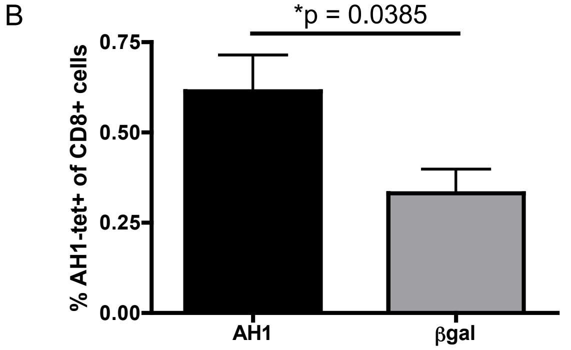

Supplementary Fig 1. Vaccination with F1A5 and WMF peptides elicits large numbers of AH1-specific T cells while small numbers of AH1-specific cells are elicited by vaccination with AH1. (a) Splenocytes from Figure 1a were stained with AH1-tet and antibodies specific for CD8 and dump molecules. The percentage of $CD8^+$ AH1-tet^+ was determined by flow cytometry and the % of maximum AH1-tet+ cells, after subtracting background AH1-tet staining in βgal vaccinated mice, was used to determine the EC_{50} values shown in Figure 1a (n = 6). Error bars represent the SEM. (b) In a separate experiment, mice vaccinated with AH1 or βgal peptides were stained as in (a) and the % of AH1-tet+ cells was determined by flow cytometry (n = 6) and compared using a Student's *t* test. A more thorough analysis of the number and function of AH1-specific cells in AH1-vaccinated mice was previously performed (Jordan 2010).

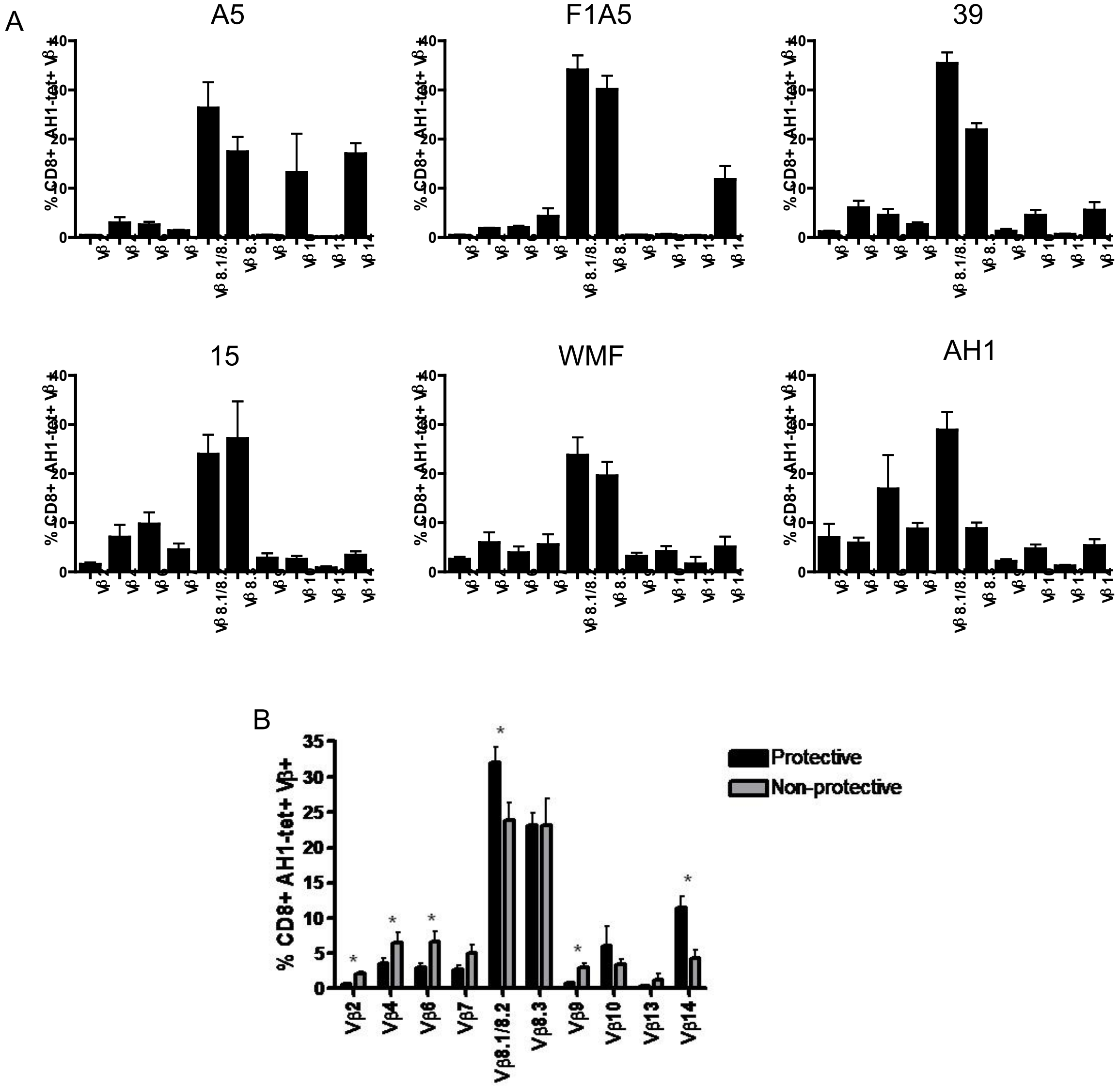

Supplementary Fig 2. Vaccination with AH1 and variant peptides elicits T cells that predominantly express TCRs encoding the Vβ8 genes. (a) Splenocytes from mice vaccinated with the indicated peptides were stained with AH1-tet for 1 h at 37°C, antibodies specific for CD8 and dump molecules, and a panel of antibodies specific for the indicated Vβ molecule were then added for additional 1 h at 4°C. The mouse TCR Vβ screening panel (besides Vβ3, Vβ5, Vβ11, Vβ12, and Vβ17 because they are not expressed by peripheral T cells of BALB/c mice, BD Pharmigen) was used for analysis of the Vβ repertoire. The percentage of $CD8^+$ AH1-tet$^+$ Vβ$^+$ was determined by flow cytometry ($n = 4$ for Vβ13 and $n = 7$ for all others). Error bars represent the SEM. (b) The data for the ineffective peptides (15 and WMF in grey) and the effective peptides (A5, 39, and F1A5 in black) were combined. The percentage of CD8+ AH1-tet+ Vβ+ for each Vβ antibody was compared using a Student's *t* test (Vβ2 $p < 0.0001$, Vβ4 $p = 0.0088$, Vβ6 $p = 0.0104$, Vβ8.1/8.2 $p = 0.0241$, Vβ9 $p < 0.0001$, Vβ14 $p = 0.0031$).

Table S1, related to Figure 4. The multiplex identifiers (MID) sequences used for high-throughput sequencing and the number of sequences analyzed from each mouse.

Vaccine[1]	MID[2]	total # of sequences analyzed
A5a	AACCATCG	20057
A5b	AACCATGC	19754
A5c	plasmid	17
A5d	plasmid	20
F1A5a	AACCGCAT	29706
F1A5b	AACCGCTA	4211
F1A5c	plasmid	22
F1A5d	plasmid	17
39a	AACCGGAA	6175
39b	AACCGGTT	5174
39c	plasmid	23
39d	plasmid	24
15a	AACCTACG	2745
15b	AACCTAGC	368
15c	plasmid	26
15d	plasmid	24
WMFa	AACCTTCC	644
WMFb	AACCTTGG	342
WMFc	plasmid	20
WMFd	plasmid	14
AH1a	AACCAACC	34977
AH1b	AACCAAGG	741
AH1c	plasmid	18
AH1d	plasmid	22

Primer Name	Primer Sequence (5' - 3')
Vβ8.1 (TRBV13-1)	CATTACTCATATGTCGCTGAC
Vβ8.2 (TRBV13-2)	CATTATTCATATGGTGCTGGC
Vβ8.3 (TRBV13-3)	TGCTGGCAACCTTCGAATAGGA
Reverse Cβ	CTTGGGTGGAGTCACATTTCTC
Internal Cβ	GAGTCACATCTCTCAGATCTT
Forward MID primer	**GCCTCCCTCGCGCCATCAG***AACCAACC*ATGGGCTGAGGCTGATCCATTA
Reverse primer	**GCCTTGCCAGCCCGCTCAG**GGCTCAAACAAGGAGACCTTGG
Forward adaptor primer	GCCTCCCTCGCGCCATCAG
Reverse adaptor primer	GCCTTGCCAGCCCGCTCAG
Vα6 Forward	ATGGCTTTCCTGGCTATTGCC
Cα Reverse	TGGCGTTGGTCTCTTTGAAG

[1]Mice were vaccinated with the indicated peptide and 4 mice were analyzed per vaccine. Mouse (a) and (b) were analyzed using high-throughput sequencing and mouse (c) and (d) were analyzed by sequencing plasmids encoding the PCR-ampified Vβ region.

[2]The MID sequences used to distinguish individual mice during the analysis of the high-throughput sequencing results are listed. We did not use MIDs during the analysis of the traditional plasmid sequencing method.

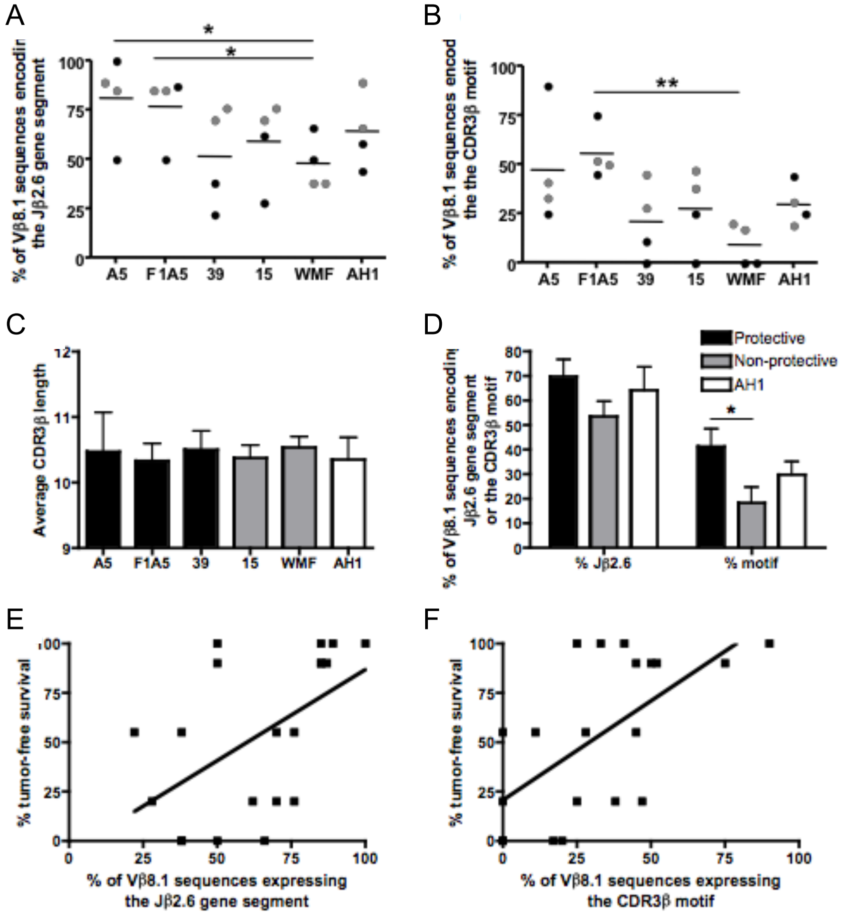

Supplemental Fig 3. The usage of Jβ2.6 and the CDR3β motif by T cells expressing Vβ8.1 TCRs correlates with tumor protection. (a) The average percentages of sequences encoding the Jβ2.6 gene segment was calculated for the sequences of the Vβ8.1-expressing TCRs from vaccinated mice (analyzed as in Fig. 5). Symbols represent individual mice analyzed by traditional sequencing methods (black) or high-throughput sequencing (grey). The bar indicates the mean and groups were compared using a Student's *t* test (* $p < 0.05$). (b) As in (a), the average percentages of sequences encoding the common CDR3β motif were calculated for each vaccine (**$p = 0.0017$). (c) The average length of the CDR3β chains was determined for the Vβ8.1 sequences. (d) The average percentage of sequences encoding the Jβ2.6 gene segment (left) or the CDR3β motif (right) were calculated for the effective (A5, F1A5, and 39 in black), ineffective (15 and WMF in grey), and native peptide (white). Error bars represent the SEM. Groups were compared using a Student's *t* test (*$p = 0.039$). (e) The frequency of sequences encoding the Jβ2.6 gene segment (x-axis, from a) was plotted versus the frequency of tumor-free survival observed for each vaccine (Jordan 2010) and analyzed using a Spearman's non-parametric correlation test ($r = 0.5856$, $p = 0.0067$). (f) As in (e), the correlation of the frequency of sequences encoding the CDR3β motif (x-axis, from b) and tumor-free survival was analyzed ($r = 0.6067$, $p = 0.0046$).
